# Tuberous sclerosis complex presenting as pulmonary lymphangioleiomyomatosis - a clinicoradiological diagnosis

**DOI:** 10.11604/pamj.2015.20.207.5490

**Published:** 2015-03-06

**Authors:** Kamini Gupta, Amit Goyal, Kavita Saggar, Avik Banerjee

**Affiliations:** 1 Department of Radiodiagnosis, Dayanand Medical College and Hospital, Ludhiana, Punjab, India

**Keywords:** Tuberous sclerosis, lymphangioleiomyomatosis, angiomyolipoma

## Abstract

Tuberous sclerosis complex (TSC) manifests predominantly as a neurocutaneous disorder. Lymphangioleiomyomatosis (LAM) is a rare pulmonary manifestation of TSC. Imaging evaluation plays an important role in the assessment of patients with tuberous sclerosis complex. In newly diagnosed patients, it helps not only to confirm the diagnosis of TSC, but also helps in identifying clinically significant complications. We describe the radiological findings in lungs and other organs in a middle aged female with TSC.

## Introduction

Tuberous sclerosis complex is an autosomal-dominant disorder characterized by the formation of hamartomatous lesions in multiple organ systems. It is the second most common neurocutaneous syndrome after neurofibromatosis type 1. Almost all patients with TSC have numerous cutaneous stigmata like hypopigmented macules, adenoma sebaceum & periungual fibromas. In addition, TSC patients develop numerous brain lesions, AMLs, LAM in the lungs, cardiac rhabdomyomas, skeletal lesions, and vascular anomalies. All these manifestations are well visualized using various imaging modalities.

## Patient and observation

**Figure 1:**
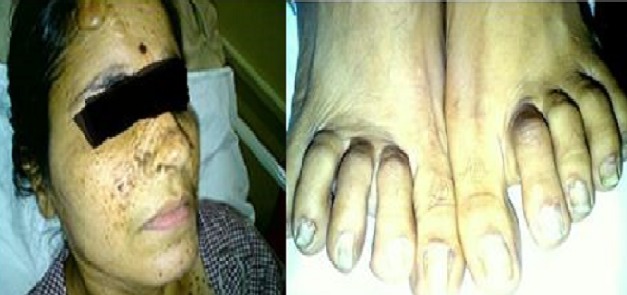
Multiple Facial Angiofibromas-Adenoma Sebaceum (left image) and Periungual Fibromas (right image)

**Figure 2:**
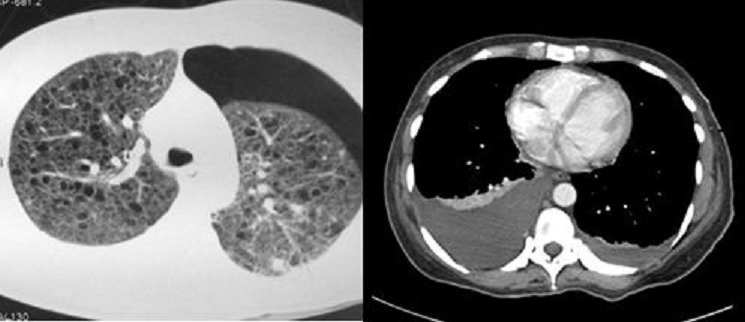
Left sided pneumothorax with multiple thin walled cystic lesions of varying sizes replacing lung parenchyma bilaterally (left image). Bilateral pleural effusion is seen (R > L) (right image)

**Figure 3:**
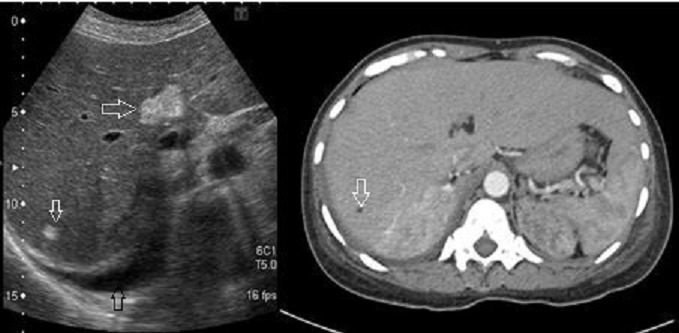
Ultrasound image shows hepatic lipomas appearing as hyperechoic lesions (white arrows). Right pleural effusion is also noted (black arrow). Corresponding CT section show fat attenuation in hepatic lesions (white arrow, right image)

**Figure 4:**
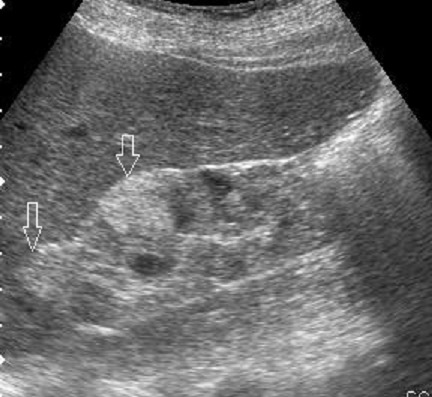
Ultrasound image shows renal cysts and angiomyolipomas seen as cystic and hyperechoic lesions in the kidneys (arrows)

**Figure 5:**
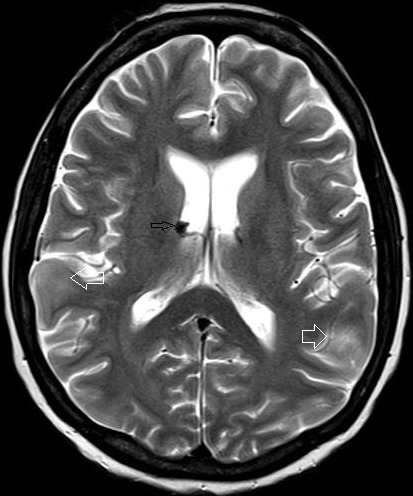
Axial section on T2W sequence shows hypointense subependymal nodules (black arrow) and hyperintense cortical tubers (white arrows)

42 year female presented with cough since 3 days, breathlessness since 2 days and chest pain since 1 day. She also had long standing history of seizures and low intelligence. On physical examination multiple adenoma sebaceum (Figure 1, left image) and periungual fibromas (Figure 1, right image) were observed on the face and feet respectively. Chest examination revealed decreased movements and reduced air entry on the left side for which CXR was done which showed pneumothorax on left side and bilateral reticulations. To characterize these lesions MDCT chest was done which revealed bilateral multiple thin walled cystic lesions of varying sizes causing partial replacement of the lung parenchyma in addition to left sided pneumothorax (Figure 2, left image). Chest tube was inserted and repeat MDCT after three days showed complete resolution of the pneumothorax. Later on patient developed bilateral pleural effusions (Figure 2, right image). Diagnostic pleural tap was done which came out to be chylous. On the basis of clinical history, physical examination and MDCT chest findings, a diagnosis of TSC was made. For the evaluation of the rest of the organs, ultrasound abdomen was done which showed few hyperechoic lesions in the liver (Figure 3, white arrows, left image) which had fat attenuation on MDCT suggesting lipomas (Figure 3, right image). Multiple heterogenous and echogenic lesions were also seen in bilateral kidneys (Figure 4, white arrows). MRI brain revealed few calcified subependymal nodules (Figure 5, black arrow). These features were consistent with a diagnosis of TSC with LAM.

## Discussion

Tuberous sclerosis complex (TSC) is the second most common phakomatosis after neurofibromatosis type 1 and is characterized by the formation of histologically benign hamartomas and low-grade neoplasms in multiple organ systems. It has a prevalence of about 1 in 6,000 newborns and affects approximately 1.5 million people worldwide, occurring in all races and both genders equally [1]. The diagnostic criteria for TSC were revised at the TSC Consensus Conference [2]. Definite TSC: Two major features or one major feature plus two minor features. Probable TSC: One major feature plus one minor feature. Possible TSC: One major feature or two or more minor features. Major and minor features have been described in Table 1.

**Table 1:** Revised diagnostic criteria for TSC at the TSC Consensus Conference by National institutes of health

Major Features	Minor Features
Facial angiofibromas or forehead plaque	Multiple randomly-distributed pits in dental enamel
Nontraumatic ungual or periungual fibromas	Hamartomatous rectal polyps
Hypomelanotic macules (three or more)	Bone cysts
Shagreen patch (connective tissue nevus)	Cerebral white matter radial migration lines
Multiple retinal nodular hamartomas	Gingival fibromas
Cortical tuber	Nonrenal hamartoma
Subependymal nodule	Retinal achromic patch
Subependymal giant cell astrocytoma	"Confetti" skin lesions
Cardiac rhabdomyoma, single or multiple	Multiple renal cysts
Lymphangioleiomyomatosis	
Renal angiomyolipoma	

The index case had five major criterias (facial angiomas, periungual fibromas, pulmonary lymphangioleiomyomatosis, angiomyolipomas and subependymal nodules) and one minor (renal cysts). Therefore, this constellation of pathognomonic clinical and imaging features was consistent with the diagnosis of TSC even in the absence of genotyping. LAM is the major lung disease associated with TSC. It was initially thought to be rare in TSC. Earlier studies estimated it to occur in 2-3% of TSC patients [3], however recently, Moss et al reported a high prevalence of 34% [4] and Costello et al reported that LAM was found in 20 (26%) of the patients [5]. Pulmonary LAM is characterized by interstitial lung injury as a result of diffuse proliferation of abnormal smooth muscles [6, 7]. Bronchovascular smooth muscle proliferation results in alveolar destruction and cystic parenchymal damage. Pulmonary lymphangioleiomyomatosis mainly affects females of reproductive age with dypsnoea and pneumthorax as the commonest clinical presentations. In a comprehensive evaluation of 35 patients with LAM, Chu et al noted dyspnoea in 83%, while 69% presented with spontanous pneumothorax. Other clinical manifestations of LAM include non-productive cough, haemoptysis, chylous pleural effusion and chylous ascites [8].

## Conclusion

LAM which occurs mainly in women of childbearing age is the major pulmonary disorder seen in TSC. History of shortness of breath or chest pain in a female particularly of child bearing age with skin lesion should alert physicians to the possibility of tuberous sclerosis. To conclude we want to highlight that MDCT findings of LAM are very characteristic and can be considered diagnostic, particularly when typical abdominal lesions are also present.
